# COVID-19 pandemic risk and probability of loan default: evidence from marketplace lending market

**DOI:** 10.1186/s40854-021-00300-x

**Published:** 2021-12-08

**Authors:** Asror Nigmonov, Syed Shams

**Affiliations:** 1 University of New South Wales, Sydney, Australia; 2 University of Southern Queensland, Darling Heights, Australia

**Keywords:** Peer-to-peer lending, COVID-19, Coronavirus, Default risk, Marketplace lending, Pandemic, FinTech, Shadow banking, E31, E43, G29, G14, O16

## Abstract

As the COVID-19 pandemic adversely affects the financial markets, a better understanding of the lending dynamics of a successful marketplace is necessary under the conditions of financial distress. Using the loan book database of Mintos (Latvia) and employing logit regression method, we provide evidence of the pandemic-induced exposure to default risk in the marketplace lending market. Our analysis indicates that the probability of default increases from 0.056 in the pre-pandemic period to 0.079 in the post-pandemic period. COVID-19 pandemic has a significant impact on default risk during May and June of 2020. We also find that the magnitude of the impact of COVID-19 risk is higher for borrowers with lower credit ratings and in countries with low levels of FinTech adoption. Our main findings are robust to sample selection bias allowing for a better understanding of and quantifying risks related to FinTech loans during the pandemic and periods of overall economic distress.

## Introduction

Diversification of risk to investors has always been the main reason and rationale behind the existence of banks (Diamond 1984). However, after the global financial crisis of 2007–2008, banks set higher standards of lending due to greater regulations. Low returns on public markets lured investors into alternative forms of investments. COVID-19 crisis could further shift investors’ preferences towards non-traditional markets, which are less liquid and transparent (Sindreu 2020). This might allow a new generation of non-banks to come of age and transform shadow banking (banking by non-banks) within the broader development of the FinTech industry. One of those businesses is marketplace lending platforms that manage to diversify risk while not holding borrower loans in their balance sheets. This method of diversification tends to protect them against borrower defaults and maintain the required level of liquidity (Cumming and Hornuf 2018).

During the last decade, the marketplace lending industry expanded outside stringent government regulations and more than doubled in size in China, the US, and the UK between 2010 and 2014 (Aveni et al. 2015). The rise of the marketplace lending market is often attributed to the stringent regulation of traditional bank lending after the global financial crisis, while the central cause of the crisis was growing credit default risk because of information asymmetry (Emekter et al. 2015). At their early stages, marketplace lending platforms essentially took a ‘passive’ role in their borrower assessment and loan provision. Individual investors mostly made investment decisions based on ‘soft’ information provided by the borrowers (Balyuk and Davydenko 2019). When the loan volumes grew larger, platforms automated investment decisions, developed individual scoring systems and algorithms distinct from banks or credit bureaus (Iyer et al. 2016; Jagtiani and Lemieux 2017). These changes transformed the lending decisions from investors to platforms and made them more similar to ordinary banks (Balyuk and Davydenko 2019; Ryan and Zhu 2018). By getting more involved in investing decisions, marketplace lending platforms should constantly improve their risk assessment for avoiding unsustainable losses (Flögel and Beckamp 2020).

The global pandemic of novel coronavirus (COVID-19) triggered extreme government measures worldwide that restricted economic activity. In the absence of a vaccine or treatment, lockdown and social distancing measures were broadly perceived as an effective way to contain the disease. These measures had a systematic impact on the financial sector and the broader economy via business closures and disruptions of the global value chain. Ongoing economic downturn as a result of the COVID-19 pandemic increases the likelihood of unsustainable losses by the marketplace lending industry. Losses in the industry are expected to increase substantially over a major economic downturn, which can quickly exhaust investor funds (Bolt et al. 2012). Unlike prior infectious disease pandemics, the COVID-19 pandemic has a vast global economic and financial impact, allowing us to study this emerging issue in a large cross-country context.

The literature related to the COVID-19 pandemic is currently in its infancy, primarily due to its ongoing and rapidly evolving nature. Nevertheless, several studies have reported the early impact of COVID-19 on banks, financial markets and the economy overall (Agosto and Giudici 2020; Auer et al. 2020; Baldwin and Di Mauro 2020; Civelek and Xiarewana 2020; Demirguc-Kunt et al. 2020a, b; Stiller and Zink 2020; Wu and Olson 2020; Bose et al. 2021). A limited number of studies examined the impact of the COVID-19 pandemic on FinTech adoption and its popularity (Fu and Mishra 2020; Najaf et al. 2021). However, no prior studies, to our knowledge, explore the implications of the COVID-19 pandemic for the risk levels and defaults in non-bank or FinTech lending markets.

To investigate the effects of COVID-19 risk on FinTech lending, we examine the likelihood of loan default due to the COVID-19 pandemic among marketplace lending loans. The central question of this study is: *How does the COVID-19 risk affect marketplace lending market defaults?* We use the loan book dataset of Mintos (Latvia) marketplace lending platform in conjunction with the borrower- and country-specific factors. Our logistic regression estimates indicate that the COVID-19 pandemic risk increases the likelihood of loan default. Specifically, the odds ratio indicates a 2.5 times higher likelihood of default in the post-pandemic period than the pre-pandemic period. We also document that marginal changes in the probability of default are 0.39–0.63% lower among the countries with high FinTech adoption than countries with low FinTech adoption. Our analysis further shows that the impact of COVID-19 risk was not solidly reflected in loan defaults until April 2020. Loans with lower ratings are also more likely to default, with a 2.30% higher likelihood of default in the post-pandemic period than the pre-pandemic period.

Our paper contributes to the existing literature in several ways. First, we provide early evidence that COVID-19 pandemic risk significantly increases the likelihood of loan default. Second, we evaluate the monthly impact of COVID-19 pandemic risk on default risk and provide evidence that the likelihood of loan defaults is highest in the month of May and June. Third, we find that borrowers credit ratings and country-level FinTech adoption act as a cushion to prevent the likelihood of loan default. Overall, we provide a unique insight into how the COVID-19 pandemic impacts the likelihood of loan default in particular. In this regard, the findings of our study might have important implications for both marketplace lending investors, platforms and policymakers.

Most of the marketplace lending platforms are currently revising their main security tool against the financial hardship, ‘provision fund’. Platforms now withhold up to 50% of investor interest income to contribute to a ‘provisional fund’ (RateSetter 2020). This type of extreme measure may be helpful to solve short-term liquidity problems but drives away yield-hungry investors in the long term. Our study allows for quantifying risks and analysing risk factors in the marketplace lending market during financial distress. Thus, it fills the gap in the existing literature by developing a cross country model tested via econometric analysis. The findings of this study in terms of COVID-19 risk can guide platform management in overcoming or controlling various risk factors. The model used in our study by including both loan and economy specific variables might serve as a framework of risk management in marketplace lending platforms. By extending the modelling and findings of the current study marketplace, lending platforms and investors can improve their risk management models. Marketplace lending platforms can improve their security mechanisms, and investors can adjust their loan portfolios.

The rest of this paper is organised as follows. Second section describes the background of the topic, and third section explains the methodology and data. Fourth section discusses the empirical findings of the study. Fifth section is the discussion and conclusion.

## Background of the topic

Several studies explore the impact of earlier pandemics on the financial sector and the economy overall. Pandemics are historically known to have a considerable economic cost attached to them that can significantly influence the financial systems (Haacker 2004; Santaeulalia-Llopis 2008; Yach et al. 2006). Goodell (2020) emphasises that the COVID-19 pandemic can be paralleled to other survivable disasters, including earthquakes, volcanic eruptions, air disasters, and terrorist attacks. COVID-19, as a survivable global pandemic, is projected to have a long-term imprint on firm financing and the cost of capital (Goodell 2020). Elnahas et al. (2018) debated that organisations located in a disaster-prone area tend to be less levered. For that reason, COVID-19 is estimated to bring together less levered principal structures.

The influence of a pandemic on the economy at the global level is undervalued. As a result, financial prudence tends to underinvest in preparation for it, which became prominent when the spending behaviours changed globally after the outbreak of the COVID-19 pandemic. Leoni (2013) studied the spread of the Human Immunodeficiency Virus in developing countries and indicated its association with deposit withdrawals. Lagoarde-Segot and Leoni (2013) forecasted that pandemics could result in a downfall in the banking industry. Moreover, pandemic affects advancing loans to the poor as investing groups, and banks get overstretched by the economic recession (Skoufias 2003). Therefore, the COVID-19 pandemic is anticipated to influence the financial sector similarly with insolvency spikes and a disproportionate reduction in the loans to the poor.

COVID-19 pandemic is distinct from earlier pandemics because of its widespread global impact on people’s health, mobility, and social welfare. The ongoing and expected decline in business revenues and household income is likely to be massive (Sadang 2020). According to the Organisation for Economic Co-operation and Development (OECD) (2020), sales volume has dropped significantly, and companies facing unsolvable financial difficulties to pay their suppliers, employees, lenders, and investors, which leads to liquidity problems. Recent studies documented the negative impact of COVID-19 on exchange rate returns, stock market volatility, firm values (Ali et al. 2020; Dawson 2020; Iyke 2020; Shen et al. 2020).

Several of the latest studies specifically addressed the role of new financial technologies to eliminate the after-effects of the COVID-19 crisis. Auer et al. (2020) state that the developments in finance could speed up the shift toward digital payments. Fu and Mishra (2020) indicate that during the COVID-19 pandemic, daily downloads of finance mobile applications increased by 24–32% among the sample of 74 countries globally. This transformation could open a divide in access to payment instruments, which could negatively impact unbanked and older consumers. The pandemic may also amplify calls to defend the role of cash and central bank digital currencies. Cecchetti and Schoenholtz (2020) emphasise the importance of an extraordinary disclosure mechanism and stress tests for restoring investor confidence in the financial sector after the COVID-19 crisis. Sahay et al. (2020) point to the positive impact of digital finance on increasing welfare and reducing inequalities in financial access following the COVID-19 crisis. They underscore that digital financial services are faster, more efficient, and typically cheaper than traditional financial services and, therefore, increasingly reaching lower-income households and small- and medium-sized enterprises (SMEs).

Agosto and Giudici (2020) perform contagion monitoring for establishing the impact of COVID-19 on digital finance in the first country affected by COVID-19, China. Agosto and Giudici (2020) highlight that in the first week of February, the cases of COVID-19 accelerated the contagion and the Shanghai Stock Exchange (SSE) composite index plummeted. They observe a negative but weak correlation between SSE returns and reported COVID-19 cases at a later stage of the epidemic. Demirguc-Kunt et al. (2020a) analyse bank stock prices worldwide to assess the impact of the COVID-19 pandemic on the banking sector. They highlight that bank stocks underperform their domestic markets and other non-bank financial firms during the COVID-19 crisis. Banks are expected to play a countercyclical lending role that put them under significant stress. At the same time, banks interact with the rest of the global financial system via multiple linkage types which particularly increases the risk of distress during the contagion. As presented by Avdjiev et al. (2019), multidimensional interlinkages between economic agents create the multi-layered network where the exposure to credit risk transfers to the non-bank private financial sector.

Li et al. (2021) show that the COVID-19 crisis adversely affects banking stability and makes it more prone to risk-taking. They also highlight that the COVID-19 outbreak and government capital injections harm the efficiency gain from shadow banking. Najaf et al. (2021) find a significant increase in FinTech loans among the US peer-to-peer lending market. However, they highlight that this increase was accompanied by a hike in unverified loans, loan terms and interest rates. Sahay et al. (2020) indicate that due to weak demand during the COVID-19 pandemic, funding conditions tighten, and transactions drop sharply. These changes in market conditions hit FinTech companies hard, especially the smaller ones and those with thinner buffers (Sahay et al. 2020).

Alternative lending markets, such as marketplace lending, mainly serve small businesses and low-income households. They are prone to use marketplace platforms as a ‘last resort’ when they lack funds and exhaust all of their available debt capacity (Walthoff-Borm et al. 2018). Moreover, marketplace lending markets tend to reduce costs and improve user experience at the expense of inaccurate credit risk measurements (Giudici et al. 2020). Accordingly, our study expects that the current pandemic negatively affects the risk incurred by marketplace lending platforms by creating a ‘bank-run’ type scenario, as projected in Peckham (2013). On the other hand, governments implemented several COVID-19 support programs to ease the financial hurdles of small enterprises and low-income households. These measures are intended to mitigate the economy’s downturn and safeguard against borrower delinquencies (Civelek and Xiarewana 2020). Gordon and Jones (2020) forecasted loan delinquency rates under the COVID-19 pandemic in three scenarios depending on the policy interventions. They predict an increase in the US loan delinquency rates from 2.3% in 2019 to 3.1% in 2021 under the baseline scenario (intermediate projection). Simultaneously, these increases might vary between 2.6 and 3.5%, depending on the policy intervention measures (Gordon and Jones 2020).

The early impact of the current pandemic on the marketplace lending market is not yet documented in empirical studies. Based on the arguments mentioned above, we predict that the COVID-19 pandemic has a negative impact on loan defaults in the marketplace lending market. At the same time, we expect the magnitude of the impact to be higher than the banking sector delinquencies. Moreover, we also expect that the pandemic has a varying impact within the marketplace lending market depending on loan ratings. The current study aims to shed more light on the early impact of COVID-19 risk on marketplace lending markets and broaden the understanding of the current market conditions.

## Methodology and data

### Regression models

We employ logit regression analysis as per Eq. (1) to measure the impact of COVID-19 pandemic risk on the likelihood of loan default risk. Equation (1) uses a binary dependent variable with a number of the country- and borrower-specific control variables. Logit regression estimates the determinants of the likelihood of loan default (*θ*_*i*_).

1 $${\theta }_{i}=\mathrm{Pr}\left({\mathrm{D}}_{i}=1|{\mathrm{Observed}}\,{\mathrm{variables}}\right)=\mathrm{Pr}(\alpha +{\beta }_{P}\mathrm{P}+{\beta }_{E}{X}_{i}^{E}+{{\beta }_{B}{X}_{i}^{B}+{\upbeta }_{L}\mathrm{L}+\varepsilon }_{i})$$

${D}_{i}$ is a binary variable representing the loan status (DEFAULT) of loan *i* that takes the value of 1 if the loan is overdue, defaulted or buyback^1^ and 0 otherwise (current or repaid). ${\beta }_{P}P$ is the variable representing the COVID-19 pandemic risk. Following the early studies of the COVID-19 pandemic (Iyke 2020; Bose et al. 2021; Nigmonov and Daradkeh 2020; Okorie and Lin 2021), we use three proxies to represent the pandemic risk: (1) a dummy variable that equals 1 for the period no later than March 11, 2020, when the World Health Organization (WHO) declared COVID-19 as the pandemic and 0 otherwise; (2) the total daily number of country-level reported cases of COVID-19 per million population and (3) the total daily number of reported COVID-19 related deaths per million population at the country level. There is no evidence from existing studies regarding the direction of the expected impact of the pandemic risk on loan defaults. However, several prior studies reported higher levels of non-performing loans (NPL) and risk exposure during the previous crises faced by financial sectors worldwide (Ari et al. 2020; Avdjiev et al. 2019; Laeven and Valencia 2013). ${\beta }_{E}{X}_{i}^{E}$ represents the vector of economy-specific control variables.^2^ We support the inclusion of economy-specific variables with existing literature on both traditional financial institutions (Ali and Daly 2010; Bofondi and Ropele 2011; Greenwald and Stiglitz 1993; Kou et al. 2021; Stiglitz and Weiss 1992) and alternative financial markets (Dushnitsky et al. 2016; Mollick 2014; Ning et al. 2015; Wang et al. 2021). The existing studies on marketplace lending extensively use borrower and loan characteristics in estimating loan defaults (Cai et al. 2016; Galema 2020; Li et al. 2018; Nigmonov et al. 2021; Serrano-Cinca et al. 2015; Wei and Lin 2017). Thus, we use the vector of borrower-specific control variables denoted as ${\beta }_{B}{X}_{i}^{B}$ in Eq. (1). We also note that marketplace lending markets are characterized by a high interdependence between loans and issuing countries. Giudici et al. (2020) suggest using borrowing companies as a vertex of correlation network for representing this interdependence and interconnections. Therefore, we use ${\upbeta }_{L}\mathrm{L}$, the vector of dummy variables, for ‘loan originator’ individual effects^3^ that in turn represent network centrality of loans.

### Data and sampling

In this study, we use the loan book database of the Mintos marketplace lending platform. The scope of this study covers the loans issued across the multiple countries of the European Union (EU). The marketplace lending market in continental Europe is experiencing high rapid growth. Countries in continental Europe have similar regulations and represent an excellent opportunity for analysing the current tendencies in marketplace lending markets.

Mintos is a marketplace for loans issued by non-banking financial institutions or the shadow banking sector. The company’s marketplace platform simultaneously lists loans from multiple lending companies, so-called ‘loan originators’. Most loans are with a buyback guarantee, a credit enhancement given by the ‘loan originator’ to the investor for a particular loan. If the loan is over 60 days late, the lending company is obligated to buy back the investment at nominal value plus accrued interest. During the market turmoil, loan originators struggle to oblige with buyback guarantees. Therefore, we classify loans that are more than 30 days late or buyback as defaults. Loan originators listed in Mintos are based in 30 countries, including 10 EU countries. Investors in the marketplace are from 66 countries, though Mintos does not disclose information about the investor categories and origination.

As the scope of our study is limited to EU countries, we restrict the database to loans issued by loan originators in EU countries. The countries included in the database are Bulgaria, Czech Republic, Denmark, Estonia, Finland, Latvia, Lithuania, Poland, Spain, Romania and the United Kingdom. These countries are similar in terms of their regulatory framework and business environment. At the same time, the inclusion of countries outside the EU would distort our analysis by complicating the comparisons and reducing the data quality. We provide the distribution of loans by loan originators in Table 12 of “Appendix 2”. During the pandemic period, Mintos discontinued some risky loans and put more stringent requirements for borrowers. This is evident from Table 12, where the number of loans and loan originators decreased during the pandemic. We address the heterogeneity of the database regarding loan originators by including the loan originator individual effects in the regression models.

**Table 1:** Breakdown of loans by rating in pre- and post-pandemic period

Rating	Pre-pandemic		Rating	Post-pandemic	
	N	%		N	%
A	3073	0.57	A	857	0.31
A−	112,652	20.91	A−	65,114	23.57
B+	194,856	36.17	B	134,486	48.69
B	17,508	3.25	B+	4565	1.65
B−	206,652	38.36	B−	70,310	25.45
C+	3489	0.65	C+	898	0.33
D	426	0.08	D	–	–
**Total**	**538,656**	**100.00**	**Total**	**276,230**	**100.00**

Total values are in bold

Table 1 provides the breakdown of loans by the borrower ratings, indicating a sharp decline in lower-rated loans. We observe that the share of loans classified as ‘B’ and ‘B−’ decreased from 3.25% and 38.36% in the pre-pandemic period to 1.65% and 25.45% in the post-pandemic period, respectively. We mitigate for different risk levels of borrowers by analysing the impact of COVID-19 risk in the breakdown of loan ratings.

The database used in this study consists of all the loans issued by Mintos loan originators based in the EU from January 2020 to June 2020. We combine each of the loans recorded in the database with the country-specific economic variables and variables representing COVID-19 risk^4^ as defined in Eq. (1). The updated database consists of 13 variables with 814,872 loan listing observations. Table 11 in ”Appendix 1” describes all variables used in this study.

Panel A of Table 2 provides the breakdown of loans by loan status for the database used in the study. Loans are classified as Current, Default, Finished (as scheduled or prematurely), Grace Period, and Late loans. However, loans classified as ‘Finished’ might be the ‘buyback’ loans, which we classify as defaults^5^ in the regression models. We provide the complete breakdown of resolved and unresolved loans for the first six months of 2020 in Panel B of Table 2. Around 65% of our database consists of loans with clear ending resolution (resolved loans), as reported in the last rows of Table 2. The share of resolved loans shrinks from around 83% in January to 3% in June 2020. We mitigate the high representation of loans with a clear ending resolution in the robustness tests section of this paper.

**Table 2:** Breakdown of loans by loan status and current resolution

Loan status	N	%	Cumulative %
*Panel A: Breakdown of loans by loan status*			
Current	182,732	22.43	22.43
Default	2	0.00	22.43
Finished as scheduled	72,916	8.95	31.38
Finished prematurely	453,359	55.65	87.03
Grace Period	12,766	1.57	88.60
Late 1–15	23,078	2.83	91.43
Late 16–30	22,378	2.75	94.18
Late 31–60	47,365	5.81	100.00
Late 60+	37	0.00	100.00
**Total**	**814,633**	**100.00**	

	N	%
*Panel B: Breakdown of loans by current resolution*		
January		
Unresolved	39,056	16.63
Resolved	195,809	83.37
Total	234,865	100.00
February		
Unresolved	55,814	25.63
Resolved	161,959	74.37
Total	217,773	100.00
March		
Unresolved	68,796	40.10
Resolved	102,764	59.90
Total	171,560	100.00
April		
Unresolved	36,509	45.69
Resolved	43,401	54.31
Total	79,910	100.00
May		
Unresolved	72,661	76.90
Resolved	21,823	23.10
Total	94,484	100.00
June		
Unresolved	15,759	96.72
Resolved	535	3.28
Total	16,294	100.00
All loans		
Unresolved	288,595	35.42
Resolved	526,291	64.58
Total	814,886	100.00

Total values are in bold

Table provides the breakdown of loans by their respective statuses. Panel A classifies all loans by the loan status. Panel B provides the breakdown of loans to resolved and unresolved loan categories for each month of 2020 and for the whole database

We report the descriptive statistics in the breakdown of pre and post-pandemic periods in Table 3. There is a statistically significant difference in loan default probabilities between these two periods. Mintos policy during the pandemic period that discontinued loans from risky borrowers lead to massive reductions in the number of issued loans and risk level of the overall loan portfolio. Mintos also complied with government regulations providing more concessions for borrowers. It is reflected in the share of loans with extended schedule (EXT_SCHED), which significantly increased during the pandemic period. We test for the possibility of default loans being bailed out by the governments in the respective robustness tests section of this paper. Table 3 also indicates significant changes in economy specific indicators and loan characteristics for which we control in all regression models.

**Table 3:** Descriptive statistics

	Pre-pandemic			Post-pandemic			Two-sample t-test	
	N	Mean	St. dev.	N	Mean	St. dev.	Mean diff.	t-stat
DEFAULT	538,656	0.110	0.312	276,230	0.074	0.262	0.036***	(51.33)
PANDEM_DUM	538,656	0.000	0.000	276,230	1.000	0.000	N/A	N/A
DAILY_CASES	226,980	0.598	2.121	276,187	24.348	40.597	− 23.750***	(− 278.40)
DAILY_DEATHS	226,980	0.005	0.044	276,187	2.053	6.059	− 2.049***	(− 161.09)
MARKET_VOL	538,656	− 0.003	0.631	276,230	0.003	1.293	− 0.005**	(− 2.59)
ESI	538,656	99.854	2.800	276,230	75.675	17.997	24.180***	(963.56)
AAR	538,656	13.066	3.284	276,230	13.289	1.960	− 0.223***	(− 32.84)
UNEMPL	538,656	6.772	4.128	276,230	8.426	4.987	− 1.653***	(− 159.19)
EXT_SCHED	538,656	0.613	0.487	276,230	0.760	0.427	− 0.146***	(− 133.74)
INTEREST	538,656	11.607	2.853	276,230	13.931	3.301	− 2.324***	(− 329.71)
LOAN_TERM	538,656	6.774	15.553	276,230	5.086	12.864	1.688***	(49.09)
AMOUNT	538,656	632.093	1134.417	276,230	546.145	968.904	85.950***	(33.97)
COLLATERAL	538,656	1.092	0.289	276,230	1.111	0.314	− 0.019***	(− 27.43)

T-statistics in parentheses. ***, **, and * represent statistical significance at the 1%, 5% and 10% levels, respectively. Variable definitions are provided in “Appendix 1”

We report a correlation matrix in Table 4 for the variables employed in the empirical analysis. The majority of the variables have a low level of statistically significant correlation with one another, as reflected in small correlation coefficients. We observe the high correlation coefficients between variables that are not used in the same model. For example, the correlation coefficient between DAILY_CASES and DAILY_DEATHS is 0.7354, indicating a strong positive correlation. We use these two variables as the different proxies of the same indicator.

**Table 4:** Correlation matrix

	DEFAULT	PANDEM_DUM	DAILY_CASES	DAILY_DEATHS	MARKET_VOL	ESI	AAR
DEFAULT	1.0000						
PANDEM_DUM	− 0.0568***	1.0000					
DAILY_CASES	0.0778***	0.3653***	1.0000				
DAILY_DEATHS	0.0316***	0.2215***	0.7354***	1.0000			
MARKET_VOL	0.0004	0.0029**	0.0020	0.0001	1.0000		
ESI	0.1338***	− 0.7298***	− 0.0904***	− 0.0840***	− 0.0023*	1.0000	
AAR	− 0.1629***	0.0364***	− 0.0790***	− 0.0119***	0.0010	− 0.1446***	1.0000
UNEMPL	0.0693***	0.1737***	0.3331***	0.2464***	0.0001	0.0558***	− 0.6595***
EXT_SCHED	0.0785***	0.1466***	0.0780***	0.0441***	0.0000	− 0.0789***	0.0402***
INTEREST	− 0.0847***	0.3431***	0.0114***	− 0.0212***	0.0018	− 0.3521***	0.2854***
LOANTERM	− 0.0389***	− 0.0543***	− 0.0976***	− 0.0664***	− 0.0007	0.0666***	0.1725***
AMOUNT	0.0413***	− 0.0376***	− 0.0651***	− 0.0459***	− 0.0007	− 0.0228***	0.1177***
RATING	− 0.0782***	− 0.1221***	− 0.0799***	− 0.0582***	0.0017	− 0.0301***	0.2552***
LOANTYPE	0.0378***	− 0.0116***	0.1075***	0.0722***	− 0.0000	− 0.0827***	− 0.1373***

	UNEMPL	EXT_SCHED	INTEREST	TERM	AMOUNT	RATING	LOANTYPE
UNEMPL	1.0000						
EXT_SCHED	0.0910***	1.0000					
INTEREST	− 0.2974***	0.4133***	1.0000				
LOANTERM	− 0.2311***	− 0.3037***	− 0.0342***	1.0000			
AMOUNT	− 0.1922***	− 0.0624***	− 0.0032**	0.0754***	1.0000		
RATING	− 0.3178***	0.3536***	0.5418***	− 0.2778***	0.0592***	1.0000	
LOANTYPE	0.0561***	0.1656***	0.3395***	− 0.2680***	0.0093***	0.5444***	1.0000

Table reports Pearson correlations. High correlations are in boldface. ***, **, and * represent statistical significance at the 1%, 5% and 10% levels, respectively (for two-tailed *p* values)

## Empirical analysis

### Baseline regression

Table 5 provides the results of the regression models with the status of loans as the dependent variable. The results show a significant impact of COVID-19 pandemic-related risk on the likelihood of loan defaults. All three proxies of the pandemic risk (PANDEMIC_DUMMY, DAILY_CASES and DAILY_DEATHS) generate significant and positive coefficients which are consistent across Models (1), (2) and (3). Specifically, the increase in COVID-19 related deaths tends to increase the likelihood of default significantly (β = 0.037). On the other hand, the number of daily reported COVID-19 cases tend to have a smaller impact on the likelihood of default with a relatively lower magnitude of a coefficient (β = 0.004). To quantify the effect of COVID-19 risk on loan status, we estimate pre- and post-pandemic default probabilities while holding all other variables constant in their mean values.^6^ Based on the baseline regression model results, the probability of default increased from 0.056 (pre-pandemic) to 0.079 (post-pandemic).

**Table 5:** COVID-19 risk and the likelihood of loan default

Variables	DV = DEFAULT	DV = DEFAULT	DV = DEFAULT
	(1)	(2)	(3)
PANDEMIC_DUMMY	0.533*** (0.006)		
DAILY_CASES		0.004*** (0.000)	
DAILY_DEATHS			0.037*** (0.001)
MARKET_VOL	0.002 (0.002)	0.001 (0.003)	0.002 (0.003)
ESI	0.031*** (0.000)	0.029*** (0.000)	0.030*** (0.000)
AAR	− 0.088*** (0.001)	− 0.086*** (0.001)	− 0.094*** (0.001)
UNEMPL	− 0.046*** (0.001)	− 0.026*** (0.001)	− 0.027*** (0.001)
COLLATERAL	− 1.342*** (0.359)	− 1.132*** (0.439)	− 1.125** (0.439)
EXT_SCHED	0.636*** (0.006)	0.726*** (0.008)	0.729*** (0.008)
INTEREST	− 0.897*** (0.012)	0.264*** (0.015)	0.352*** (0.015)
LOANTERM	− 0.093*** (0.003)	− 0.087*** (0.004)	− 0.083*** (0.004)
AMOUNT	0.192*** (0.002)	0.225*** (0.003)	0.226*** (0.003)
*LOAN TYPE*			
Business loan	0.670** (0.277)	1.342*** (0.373)	1.345*** (0.374)
Car loan	1.399*** (0.369)	1.547*** (0.470)	1.540*** (0.471)
Pawnbroking loan	1.694*** (0.367)	2.256*** (0.469)	2.267*** (0.470)
Personal loan	1.239*** (0.111)	1.551*** (0.175)	1.537*** (0.176)
Short-term loan	0.953*** (0.110)	1.102*** (0.175)	1.093*** (0.176)
Intercept	− 2.468*** (0.386)	− 5.883*** (0.479)	− 6.070*** (0.480)
Loan originator individual effects	Yes	Yes	Yes
LR chi2	68,062.632	59,563.239	58,885.153
Prob > chi2	0.000	0.000	0.000
Pseudo-R-squared	0.131	0.175	0.173
N	814,872	503,167	503,167

Table presents the results of logit regression analysis for the likelihood of loan default (DEFAULT). Number of loans analysed: 814,872. Current or repaid: 735,387 (90.25%). Default, late or buyback: 79,485 (9.75%). Refer to Table 11 in “Appendix 1” for the description of variables. All model specifications employ robust standard errors in parentheses (*p < 0.10, **p < 0.05, ***p < 0.01)

### Additional analyses

Another specific aspect of our sample is that it incorporates diverse countries regarding their efficiency of the credit market and FinTech development. On the other hand, these countries are similar in terms of their geographical location and operate under the EU jurisdiction. This unique aspect of our database creates an opportunity to explore the impact of the COVID-19 pandemic on defaults in the breakdown of countries’ FinTech development. Specifically, we can examine whether FinTech has suppressed the effect of the COVID-19 pandemic on borrower creditworthiness.

Table 6 provides the breakdown of our baseline model in panels based on the level of FinTech adoption in individual countries. We divide our database based on the Global Fintech Index reported by Findexable (2019), which provides a snapshot of local business infrastructure and FinTech ecosystem quality. Panels reported in Table 6 are based on subsamples of countries that are reported to have higher/lower than the median Global FinTech Index. We run the same baseline logit regression model on these two subsamples. We observe that the impact of the COVID-19 pandemic on loan defaults has been more severe in Panel B of Table 6 (lower than median Global FinTech Index). This finding is reflected in the coefficients of COVID-19 proxies that are higher for Panel B of Table 6.

**Table 6:** COVID-19 risk and the likelihood of loan default: the role of FinTech adoption

Variables	DV = DEFAULT		
	(1)	(2)	(3)
*Panel A: high FinTech adoption*			
PANDEMIC_DUMMY	0.267*** (0.007)		
DAILY_CASES		0.003*** (0.000)	
DAILY_DEATHS			0.025*** (0.001)
Loan originator individual effects	Yes	Yes	Yes
Controls	Yes	Yes	Yes
LR chi2	75,221.734	58,608.936	58,155.240
Prob > chi2	0.000	0.000	0.000
Pseudo-R-squared	0.171	0.191	0.189
N	588,385	415,370	415,370
*Panel B: low FinTech adoption*			
PANDEMIC_DUMMY	0.392*** (0.023)		
DAILY_CASES		0.009*** (0.001)	
DAILY_DEATHS			0.216*** (0.057)
Loan originator individual effects	Yes	Yes	Yes
Controls	Yes	Yes	Yes
LR chi2	10,252.819	5555.505	5532.129
Prob > chi2	0.000	0.000	0.000
Pseudo-R-squared	0.164	0.219	0.218
N	226,487	87,783	87,783

Table reports the results for two panels. Panel A reports the findings of logit regression analysis for countries with high levels of FinTech adoption. Panel B reports the same findings for countries with low levels of FinTech adoption. The panels are based on countries’ FinTech Development Index (Findexable 2019) being higher/lower than the global median. All model specifications employ robust standard errors in parentheses (*p < 0.10, **p < 0.05, ***p < 0.01)

**Fig. 1:**
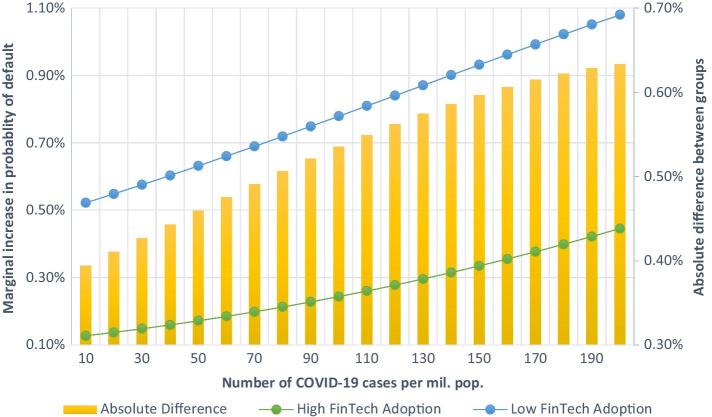
Change in the probability of default for incremental change in COVID-19 cases (by countries with high and low FinTech adoption). *Note*: Figure presents marginal changes in the probability of default for countries with high and low FinTech adoption (line plot, left axis). Calculations of marginal changes are based on the coefficients of logit regression reported in Table 6. The yellow bar plot presents the absolute differences in marginal changes between the two groups (right axis)

However, regression models with binary responses are not directly comparable (Kuha and Mills 2020). Therefore, we calculate the change in probability of default for incremental changes in COVID-19 cases. We predict the model using the logit function with estimated coefficients and hold all variables in their mean values (other than reported COVID-19 cases per million population). We report the respective marginal changes in Fig. 1 for ‘Low/High FinTech Adoption’ subsamples. Figure 1 indicates that the marginal changes in the probability of default were higher in countries with low levels of FinTech adoption. The absolute difference between the two subsamples ranges between 0.39 and 0.63% for each additional ten daily reported cases of COVID-19 per pillion population (reported as a bar plot in Fig. 1).

Prior studies indicate that FinTech might benefit small businesses and low-income households through better access to finances (Ozili 2018; Demirguc-Kunt et al. 2018). In addition, early studies of the COVID-19 pandemic also highlight the acceleration in financial technologies that can be further leveraged to overcome economic fallout from the pandemic (Arner et al. 2020; Civelek and Xiarewana 2020). In support of these early speculations, our study is the first to empirically indicate that FinTech helps curb the impact of the COVID-19 pandemic on borrower defaults.

We also analyse the monthly impact of COVID-19 risk on loan default risk using the subsamples for each month of 2020 (February to June). Our database is sorted by the last payment date of the loan and reflects borrowers’ instant exposure to pandemic risk. We report this additional analysis based on subsamples of the database in Table 7. Loan defaults did not solidly reflect the impact of COVID-19 risk until April 2020. Pandemic induced loan default risk is higher in May to June of 2020 as the coefficients for COVID-19 pandemic risk are consistently significant and positive. The early studies show that the COVID-19 pandemic risk creates substantial stress in financial markets despite some improvements in liquidity conditions (Agosto and Giudici 2020; Demirguc-Kunt et al. 2020a; Kargar et al. 2020). Using monthly analysis, we indicate that those conditions further deteriorated and significantly affected the default risk during May and June 2020.

**Table 7:** COVID-19 risk and the likelihood of loan default: monthly subsamples

Variables	DV = DEFAULT	
	(1)	(2)
*Panel A: February listings*		
DAILY_CASES	− 0.024*** (0.004)	
DAILY_DEATHS		− 0.901*** (0.137)
Controls	Yes	Yes
LR chi2	22,516.483	22,544.843
Prob > chi2	0.000	0.000
Pseudo-R-squared	0.250	0.250
N	125,546	125,546
*Panel B: March listings*		
DAILY_CASES	0.001*** (0.000)	
DAILY_DEATHS		0.007*** (0.001)
Controls	Yes	Yes
LR chi2	17,501.061	17,280.770
Prob > chi2	0.000	0.000
Pseudo-R-squared	0.101	0.100
N	200,508	200,508
*Panel C: April listings*		
DAILY_CASES	0.000** (0.000)	
DAILY_DEATHS		− 0.000 (0.001)
Controls	Yes	Yes
LR chi2	29,894.550	29,888.341
Prob > chi2	0.000	0.000
Pseudo-R-squared	0.208	0.208
N	162,099	162,099
*Panel D: May listings*		
DAILY_CASES	0.006*** (0.000)	
DAILY_DEATHS		0.061*** (0.001)
Controls	Yes	Yes
LR chi2	26,130.763	26,350.926
Prob > chi2	0.000	0.000
Pseudo-R-squared	0.348	0.351
N	176,047	176,047
*Panel E: June listings*		
DAILY_CASES	0.008*** (0.000)	
DAILY_DEATHS		0.087*** (0.002)
Controls	Yes	Yes
LR chi2	12,940.095	13,211.923
Prob > chi2	0.000	0.000
Pseudo-R-squared	0.428	0.437
N	123,614	123,614

Table presents the results of regression analyses based on five panels (for each month from February to June 2020). Results are for logit regression analysis for the likelihood of loan default (DEFAULT). All model specifications employ robust standard errors in parentheses (*p < 0.10, **p < 0.05, ***p < 0.01)

We highlight that the loan quality significantly increased during the pandemic because of the platform’s active role in managing the loans. One can argue that increased loan ratings mechanically cause changes in default or overdue loans. Accordingly, Table 8 provides the results of the analysis using the subsamples based on loan ratings. We divide the database into three groups: Panel A ('A' & 'A−' rated loans), Panel B ('B+' & 'B' rated loans) and Panel C ('B−', 'C+' & 'D’ rated loans). All but one regression model reported in Table 8 generate significant positive coefficients for the proxies of COVID-19 risk. The analysis shows that the COVID-19 pandemic risk increases the probability of loan default irrespective of the loan ratings. Thus, regardless of the loan ratings, the likelihood of default risk increases for the whole loan portfolio of the Mintos marketplace during the pandemic.

**Table 8:** COVID-19 risk and the likelihood of loan default: rating subsamples

Variables	DV = DEFAULT		
	(1)	(2)	(3)
*Panel A: 'A' & 'A*−*' rated loans*			
PANDEMIC_ DUMMY	0.491*** (0.024)		
DAILY_CASES		0.013*** (0.001)	
DAILY_DEATHS			− 0.170*** (0.061)
Loan originator individual effects	Yes	Yes	Yes
Controls	Yes	Yes	Yes
LR chi2	6449.866	3820.249	3750.524
Prob > chi2	0.000	0.000	0.000
Pseudo-R-squared	0.158	0.194	0.190
N	181,696	86,761	86,761
*Panel B: 'B*+*' & 'B' rated loans*			
PANDEMIC_DUMMY	0.078*** (0.009)		
DAILY_CASES		0.003*** (0.000)	
DAILY_DEATHS			0.029*** (0.001)
Loan originator individual effects	Yes	Yes	Yes
Controls	Yes	Yes	Yes
LR chi2	60,876.506	49,092.062	49,065.819
Prob > chi2	0.000	0.000	0.000
Pseudo-R-squared	0.178	0.187	0.187
N	351,415	297,125	297,125
*Panel C: ‘B*−*’, ‘C*+*’ and ‘D’ rated loans*			
PANDEMIC_DUMMY	0.489*** (0.020)		
DAILY_CASES		0.014*** (0.001)	
DAILY_DEATHS			0.176*** (0.026)
Loan originator individual effects	Yes	Yes	Yes
Controls	Yes	Yes	Yes
LR chi2	15,205.346	6110.321	6001.120
Prob > chi2	0.000	0.000	0.000
Pseudo-R-squared	0.206	0.197	0.193
N	281,744	119,262	119,262

Table presents the results of regression analyses based on three panels (by loan ratings). Results are for logit regression analysis for the likelihood of loan default (DEFAULT). All model specifications employ robust standard errors in parentheses (*p < 0.10, **p < 0.05, ***p < 0.01)

**Fig. 2:**
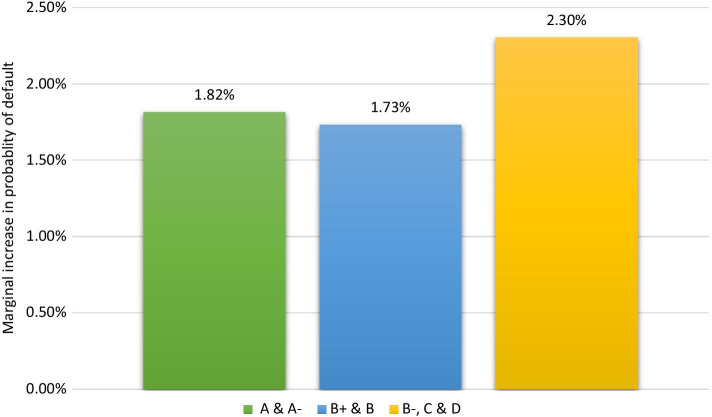
Marginal change in the probability of default during the pandemic period (by loan ratings). *Note*: Figure presents marginal increases in the probability of default during the pandemic period based on loan ratings. Calculations of marginal changes are based on the coefficients of logit regression as reported in Table 8

To assess the change in default risk based on loan ratings, we estimate the marginal magnitude of COVID-19 risk for three rating subgroups. We hold all the control variables at their mean values and estimate the change in the likelihood of default in the post-pandemic period compared with the pre-pandemic period. We base our parameters on the findings reported in Table 8. By estimating the change in the likelihood of default for three rating subgroups, we find that the likelihood of default increase by 1.82% and 1.73% for ‘A & A−’ and ‘B+ & B’ rating subgroups, respectively (Fig. 2). The change in the likelihood of default is greater for the ‘B−, C & D’ rating subgroup. On average, loans in this category are 2.30% more likely to default in the post-pandemic period than the pre-pandemic period.

**Fig. 3:**
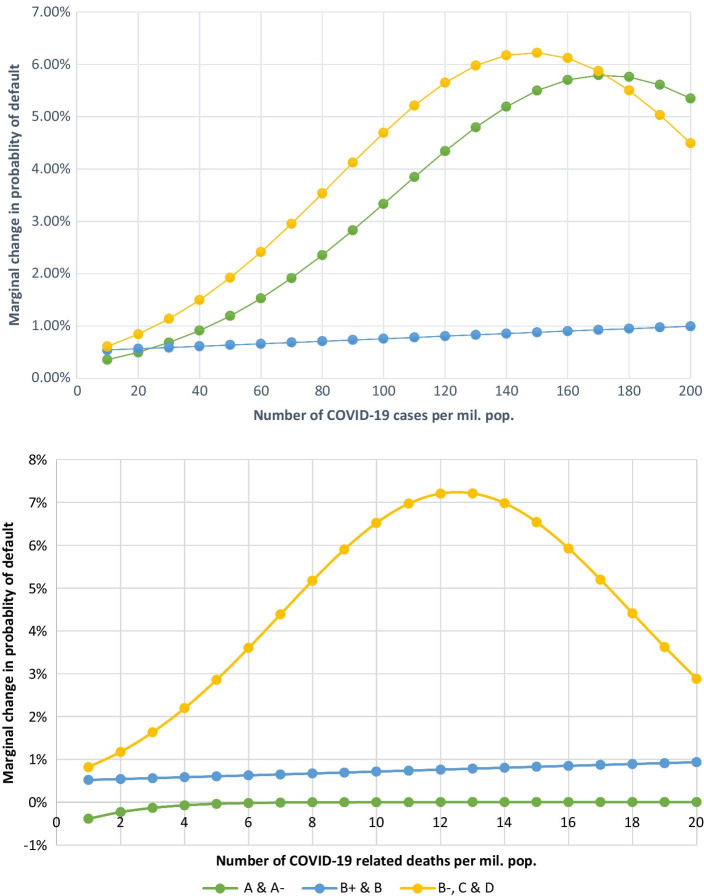
Change in the probability of default for incremental change in COVID-19 cases and deaths (by loan ratings). *Note*: Figure presents marginal changes in the probability of default based on loan ratings. Calculations of marginal changes are based on the coefficients of logit regression as reported in Table 8. Marginal changes are for each incremental change in COVID-19 cases and deaths per million population in each country

We also estimate the change in the likelihood of default for each incremental increase in COVID-19 related cases and deaths. Figure 3 reports these changes in the breakdown of borrower rating groups. We observe that B+ and B rated loans are less affected by the changes in COVID-19 related cases and deaths. On the other hand, lower-rated loans (‘B−, C & D’ category) are affected by a greater magnitude under each incremental change in COVID-19 cases and deaths. The change in the likelihood of default also decreases after achieving a certain level of peak cases and deaths. Loans with high ratings behave differently when analysed under the incremental changes in COVID-19 cases compared with COVID-19 deaths. The likelihood of default considerably increases for ‘A & A−’ rated loans under each incremental change in COVID-19 cases. On the contrary, the loans under this classification remain relatively stable in the case of COVID-19 related deaths.

Early studies of Najaf et al. (2021) indicate that risk profiles of borrowers in the US marketplace lending market significantly deteriorated during the COVID-19 pandemic. Our study highlights the same tendency for the European marketplace lending market. Further, the findings of our study indicate that the lower risk profiles transferred into higher defaults with the severity of the spread of the COVID-19 pandemic. Therefore, our results related to loan ratings might have important implications and open room for further research, which we discuss in the last section of this paper.

### Robustness tests

Our sample for the analysis comprises of heterogeneously distributed observations across loan originators. For instance, listings are overrepresented by loan originators based in Latvia, Poland and Spain.^7^ The database also contains disproportionately large amounts of observations for the pre-pandemic period. These features of the database potentially create complications related to sample selection bias. Another potential problem with our sample database is the heterogeneous distribution of loans with a clear ending resolution. This potentially creates a misrepresentation of the sample selection as the loans included in the earlier periods may be defaulted or buyback loans. These loans might be affected by a favourable environment before the pandemic induced turmoil and impede the assessment of COVID-19 risk.

To address these issues, we employ four different procedures. Firstly, we create the subsample consisting of only three countries with the highest number of observations. Secondly, we use a random bootstrap sampling^8^ technique to obtain robust estimates of the relevant coefficients. This method reduces the sampling bias and warrants that our estimates are not affected by the under-weighting or over-weighting of a certain group of observations (Chernick and LaBudde 2014; Tibshirani and Efron 1993). Thirdly, we create a subsample, including only unresolved loans that contain 288,595 loans. Fourthly, we apply the Heckman selection model for sample selection to the binary regression model. We use a binary dependent variable equal to 1 if the loan is ‘resolved’ having a clear outcome and 0 otherwise. The selection equation is estimated from a much larger sample, including the loans issued before 2020 (N = 28,054,125). We instrumentalise the selection in the sample with loan-specific variables, including the loan rating.

Table 9 reports the results of logistic regression after controlling for the above selection bias techniques. We observe that the results are identical to the findings of baseline regressions reported in Table 5 in terms of the coefficient signs and significance. The results are generally robust to all four specifications and similar to the ones reported in baseline regression. We conclude that the detected impact of COVID-19 risk is almost not affected by the selecting mechanisms to construct our sample.

**Table 9:** COVID-19 risk and the likelihood of loan default: testing for sampling bias

Variables	DV = DEFAULT		
	(1)	(2)	(3)
*Panel A: Three big countries by the number of observations*			
PANDEMIC_ DUMMY	0.405*** (0.008)		
DAILY_CASES		0.004*** (0.000)	
DAILY_DEATHS			0.045*** (0.001)
Loan originator individual effects	Yes	Yes	Yes
Controls	Yes	Yes	Yes
Pseudo-R-squared	0.210	0.235	0.237
N	680,694	390,133	390,133
*Panel B: Bootstrap sampling*			
PANDEMIC_ DUMMY	0.555*** (0.007)		
DAILY_CASES		0.004*** (0.000)	
DAILY_DEATHS			0.036*** (0.001)
Loan originator individual effects	Yes	Yes	Yes
Controls	Yes	Yes	Yes
Pseudo-R-squared	0.129	0.173	0.170
N	814,872	503,167	503,167
*Panel C: Only unresolved loans*			
PANDEMIC_DUMMY	0.387*** (0.010)		
DAILY_CASES		0.007*** (0.000)	
DAILY_DEATHS			0.067*** (0.001)
Loan originator individual effects	Yes	Yes	Yes
Controls	Yes	Yes	Yes
Pseudo-R-squared	0.274	0.345	0.347
N	288,595	213,036	213,036
*Panel D: Heckman correction*			
PANDEMIC_DUMMY	0.088*** (0.001)		
DAILY_CASES		0.001*** (0.000)	
DAILY_DEATHS			0.003*** (0.000)
Loan originator individual effects	Yes	Yes	Yes
Controls	Yes	Yes	Yes
N	814,886	503,167	503,167

Table presents the results of regression analyses based on four panels. Panel A results are for logit regression analysis for the likelihood of loan default (DEFAULT) with the sample consisting of only three countries with the highest number of observations. Panel B reports the results after the application of bootstrap sampling with stratified sampling based on loan originators and each month of 2020. Panel C results are for logit regression analysis with the sample consisting of only unresolved loans. Panel D reports the results after the application of the Heckman selection model, where the selection in the sample is instrumentalised with loan amount and rating. All model specifications employ robust standard errors in parentheses (*p < 0.10, **p < 0.05, ***p < 0.01)

We also indicate the impact of several government interventions in the form of COVID-19 moratoria and public guarantees during the period under consideration [European Banking Authority (EBA) 2020]. Due to the considerable financial support from various governments during the pandemic period, there is a possibility of default loans being effectively bailed out by the governments. In fact, Mintos continued serving late loans but fell under the government moratoria and public guarantee. However, the platform classifies these loans as overdue loans instead of writing them off (as default or buyback) from the books (Mintos 2020).

To mitigate the impact of government interventions, we estimate two separate regression models with different dependent variables than the baseline regression. First, we run an ordered logit model in which the dependent variable is the status of the unresolved loan (‘current’, ‘in grace period’ or late loans^9^). Accordingly, the dependent variable (LOANSTATUS) takes one of the six values, and the regression sample consists of only unresolved loans. Second, we run the logit regression with the dependent variable, which takes the value of 1 if the loan is classified as default or buyback and 0 otherwise.

The results are generally robust to this change in the regression method with the estimation results from Model (2) and (3) of Table 10 Panel A, similar to those reported in baseline regression. The log-likelihood of loan status transferring from lower group to one level higher group (e.g. from ‘current’ status to ‘in grace period’ status) increases with an increase in COVID-19 related daily cases and deaths. In Model (1) of Table 10 Panel A, the dummy variable representing the impact of the COVID-19 pandemic is negative on loan status. A possible explanation could be that most loans from the early months of 2020 are already resolved, coinciding with the pre-pandemic period. This result might also indicate some impact of government interventions as more borrowers apply for extensions. Nevertheless, we observe from Panel B of Table 10 that the coefficients for the variables representing COVID-19 risk remain to be significantly positive. Thus, there is some indication that government interventions only delayed the occurrence of defaults in the marketplace lending market. Our additional analyses mostly validate the baseline regression model results and provide some further insights into the impact of COVID-19 risk on loan defaults. However, the findings related to the impact of government interventions require further analyses that are duly mentioned in the last section of this paper.

**Table 10:** COVID-19 risk and the likelihood of loan default: Robustness tests for government intervention

Variables	DV = LOANSTATUS		
	(1)	(2)	(3)
*Panel A: Ordered logit model*			
PANDEMIC_DUMMY	− 0.165*** (0.010)		
DAILY_CASES		0.007*** (0.000)	
DAILY_DEATHS			0.074*** (0.001)
Loan originator individual effects	Yes	Yes	Yes
Controls	Yes	Yes	Yes
LR chi2	90,191.487	60,621.580	59,476.467
Prob > chi2	0.000	0.000	0.000
Pseudo-R-squared	0.189	0.213	0.221
N	288,356	212,917	212,917

Variables	DV = ONLYDEFAULTS		
	(1)	(2)	(3)
*Panel B: Only default loans as dependent variable*			
PANDEMIC_DUMMY	0.687*** (0.007)		
DAILY_CASES		0.007*** (0.000)	
DAILY_DEATHS			0.061*** (0.001)
Loan originator individual effects	Yes	Yes	Yes
Controls	Yes	Yes	Yes
LR chi2	54,149.904	39,331.896	37,516.142
Prob > chi2	0.000	0.000	0.000
Pseudo-R-squared	0.150	0.160	0.153
N	814,872	503,153	503,153

Table reports the results for two panels. Panel A reports the findings of ordered logit regression analysis for the loan status (LOANSTATUS) with the sample consisting of only unresolved loans. The dependent variable is an ordered dependent variable that takes one of the six values (current, in grace period, 1–15 days late, 16–30 days late, 31–60 days late and 60+ days late). Panel B reports the logit regression findings with only the default loans (ONLYDEFAULTS) as the dependent variable. The dependent variable takes the value of 1 if the loan is classified as default or buyback and 0 otherwise. All model specifications employ robust standard errors in parentheses (*p < 0.10, **p < 0.05, ***p < 0.01)

## Discussion and conclusion

### Contributions

The rapidly growing FinTech industry first-time witnesses global pandemic since their revolution of platform-based financing. As the current COVD-19 pandemic related crisis progresses into the later stage, financial hardship experienced by households, businesses, and public sector organisations might also transfer into a more severe stage. As one of the risky financing sectors, marketplace lending might experience a wave of defaults during 2021. This stream of defaults tends to impact the resilience of the industry and force platforms to reconsider their risk management models. On the other hand, the pandemic can help transform ‘shadow banking’ with an extensive emphasis on alternative lending practices (Sindreu 2020). As one of the prominent facets of alternative lending, the marketplace lending market may become mainstream from its current niche position.

Our study explores the implication of COVID-19 pandemic risk on the likelihood of marketplace loan defaults using the loan book database of Mintos. Prior studies have not yet assessed the default risk within the context of the COVID-19 pandemic. Due to the pandemic’s ongoing and rapidly evolving nature, the implication of COVID-19 pandemic-related risk and its related financial consequences are little understood.

This study documents evidence of the early detrimental impact of the pandemic induced economic turmoil on the marketplace lending market. We provide the first evidence that COVID-19 pandemic risk considerably increases the likelihood of loan defaults in the Mintos marketplace lending platform. By employing the logit regression model, we estimate that the likelihood of default, on average, increases from 0.056 in the pre-pandemic period to 0.079 in the post-pandemic period. Pandemic induced loan default risk reached its highest magnitude during May and June of 2020. We empirically document higher levels of FinTech adoption reduces adverse effects of COVID-19 on the probability of default. We also highlight a significant difference in the marginal impact of COVID-19 risk among the loan rating subgroups. Borrowers with lower credit ratings are most affected during the pandemic period.

During the pandemic induced turmoil, the findings related to COVID-19 risk have important implications. Early studies indicate that the COVID-19 pandemic has a detrimental impact on the financial sector performance, liquidity and risk profile (Baig et al. 2020; Demirguc-Kunt et al. 2020b; Najaf et al. 2021). A recent study by Ari et al. (2020) indicates that banking sector NPLs peaked at about 20% of total loans on average during the past crises since 1900. We suggest that current pandemic-related risk creates not only a liquidity crisis but also an underperformance for non-bank financial institutions. In fact, households and businesses experience financial distress, which increases default risk in one of the risky sectors of financing.

Our study is the first one that reveals how borrowers’ creditworthiness behaved during the pandemic’s early period. We also provide evidence that FinTech has suppressed the impact of the COVID-19 pandemic on borrower distress. Thus, this study allows for a better understanding and quantifying risks related to FinTech loans during the pandemic and periods of overall economic distress. It fills the gap in the existing literature by developing a cross country model that is tested via econometric analysis. Our insights into marketplace lending contribute to the literature by providing a deeper understanding of borrower behaviour under financial distress. This study’s findings in terms of COVID-19 risk can guide platform management in overcoming or controlling various risk factors. At the same time, the findings related to FinTech adoption might be used by governing bodies for further adoption and regulation of the FinTech sector. The model used in our study by including both loan and economy specific variables might serve as a framework of risk management in marketplace lending platforms.

Considering beyond borrower-specific factors, these models allow for comprehensive estimation of credits risk, borrower ratings, informing investors about potential risk levels and setting up the ‘provision fund’. Based on the same evidence, forecasting mechanisms may be put in place for mitigating risk factors in a way that were not possible before. For instance, the sensitivity of credit risk to external factors is reflected in variable coefficients in regression analyses. These coefficients highlight the sensitivity of default risk to the changes in a pandemic or its severity (reflected in the number of reported cases and deaths). These coefficients may be used for stress testing of marketplace lending portfolios under certain conditions, such as decreased consumer confidence.

### Limitations and future research avenues

As one of the early studies on the COVID-19 pandemic, our study has some limitations, which may provide important avenues for future research. First, we find some discrepancies in the analysis of COVID-19 risk in the breakdown of loan ratings. Higher rated loans (‘A&A−’) are affected by COVID-19 risk by a higher magnitude when measured by the case numbers than death numbers (Fig. 2). A possible explanation for this can be that deaths are the lagging indicator. Cases usually indicate early changes in the pandemic turmoil where both investors and borrowers are keen to cash in, while the true impact of the pandemic related disruptions did not hit the economy (Langreth et al. 2020). The number of COVID-19 related deaths, lagging a couple of weeks behind, might represent the later period of the pandemic related insolvency of borrowers. Further ascertaining this hypothesis is out of this study’s scope and requires further disclosure from the marketplace lending platforms. Future research might examine this specific aspect of loan lending if more borrower information becomes available.

Second, we focus on one specific marketplace lending platform (Mintos). Although our study includes the loans issued by various loan originators located in multiple countries of Continental Europe, the generalisation of our findings might be problematic. For example, other platforms might practice different selection mechanisms or loan risk assessment methods. Therefore, future research could test whether our findings are generalisable to marketplace lending markets in other geographical areas or other non-bank lending markets. For instance, future studies can analyse marketplace lending markets in America and Asia with regards of their exposure to COVID-19 pandemic risk.

Third, marketplace lending markets are characterized by a high interdependence between various players of the market like borrowers, platforms and issuing countries. Therefore, it is important to include financial contagion risk in the measurement of default risk probability. Future studies can analyse the default risk from the perspective of contagion risk. In this regard, theoretical considerations of the full network contagion model (Avdjiev et al. 2019) or the network-based credit risk models (Giudici et al. 2020) can be used for further extension of the modelling of this study. Nevertheless, given that Mintos is one of the most diverse and long-serving marketplace lenders in Continental Europe, it is unlikely that other platforms can match the same market coverage. Still, it would be useful to examine how country-level characteristics or interplatform competition could influence the credit risk incurred by investors.

Fourth, we consider the FinTech adoption as a cushion for curbing the negative impact of COVID-19 risk on marketplace lending market defaults. We divide our database into two separate groups based on FinTech Development Index (Findexable 2019) and calculate marginal changes in default probabilities in these two groups. However, we believe that this issue can be further extended in future studies. For instance, further analysis might estimate the indirect impact of the COVID-19 pandemic via FinTech adoption. In doing so, future studies can use mediation analysis and use alternative proxies for FinTech adoption. Future studies can also consider the role of digital finance, credit market efficiency and financial inclusion in mediating the impact of COVID-19 on defaults.

Finally, we have to note the limited scope of this study regarding the long-term impact of COVID-19 risk on the marketplace lending market. This study analysed the impact of the pandemic based on the database with a limited time span, essentially during the first wave of the pandemic. However, extending our study to the impact of subsequent second and third waves of the pandemic requires different set of conceptual framework and modelling. Particularly, after the introduction of vaccines the emphasis is shifted from case and death numbers to vaccination and hospitalization rates. We also believe that the full impact of the pandemic to credit risk can be observed by the end of 2021. By this time, it is expected that various government-imposed restrictions are lifted, and short-term liquidity problems of businesses and households are transferred into insolvency. Therefore, future studies can repeat this exercise in future when the longer timespan data become available.

## Data Availability

Data is available on request.

## Appendix 1

See Table 11.

**Table 11:** Description of variables

Variable	Description of variable	Source
*Dependent variables*		
DEFAULT	Current status of individual loan. Dummy variable equal to 1 if the loans is overdue, defaulted or buyback and 0 otherwise (current or repaid)	*Mintos*
*Independent variables, pandemic indicator variables*		
PANDEM_DUM	Dummy variable equal to 1 for the dates later than March 11, 2020 (The date WHO declared COVID-19 as pandemic) and 0 otherwise	*World Health Organization (2020)*
DAILY_CASES	Number of reported daily cases of COVID-19 per million population in country *i* at time *t* (daily observations, log values)	*World Health Organization (2020), Johns Hopkins University & Medicine (2020)*
DAILY_DEATHS	Number of reported daily COVID-19 related deaths per million population in country *i* at time *t* (daily observations, log values)	*World Health Organization (2020), Johns Hopkins University & Medicine (2020)*
*Independent variables, macroeconomic and country-specific variables*		
ESI	The EU Economic sentiment indicator (composite measure, average = 100, log values)	*Full business and consumer survey results, European Commission* https://ec.europa.eu/info/business-economy-euro/indicators-statistics/economic-databases/business-and-consumer-surveys_en
MARKET_VOL	Change in daily stock market index values of country i at time t (percentage points)	Yahoo.Finance, https://finance.yahoo.com/world-indices/
AAR	Annualised agreed rate by credit and other institutions in country *i* at time *t* (monthly, percentage points	*ECB Statistical Data Warehouse *http://sdw.ecb.europa.eu/
UNEMPL	Unemployment rate for each country (Monthly, seasonally adjusted, percentage points)	*OECD (2020), Unemployment rate (indicator). *https://doi.org/10.1787/b86d1fc8-en* (Accessed on 14 June 2020)*
*Independent variables, loan-specific variables*		
EXT_SCHED	Dummy variable representing the restructuring of a loan. Equal to 1 if the original maturity date of the loan has been increased by more than 60 days, 0 otherwise	*Mintos*
COLLATERAL	Dummy variable representing the loan type in terms of a provision of collateral. Equal to 1 if the loan is collateralised, 0 otherwise	*Mintos*
INTEREST	Maximum interest rate accepted in the loan application (%, log values)	*Mintos*
LOAN_TERM	Duration of loan (in months, log values)	*Mintos*
AMOUNT	Value of individual loan (log values)	*Mintos*
RATING	‘Mintos Rating’ issued by the rating model ranging between A+ (1) and D (7)	*Mintos*
LOANTYPE	The loan type: 1-Business Loan, 2-Car Loan, 3-Invoice Financing, 4-Pawnbroking Loan, 5- Personal Loan, 0-Other	*Mintos*

## Appendix 2

See Table 12.

**Table 12:** Breakdown of loans by loan originators in pre- and post-pandemic period

Country	Loan originator	Pre-pandemic		Post-pandemic	
		N	%	N	%
Bulgaria	CashCredit	7861	1.46	3290	1.19
Bulgaria	Credissimo	6549	1.22	1850	0.67
Bulgaria	ITF Group	317	0.06	190	0.07
Bulgaria	Mogo	312	0.06	72	0.03
Bulgaria	StikCredit	4598	0.85	1800	0.65
Czech Republic	Creamfinance	1910	0.35	1120	0.41
Czech Republic	Creditstar	2350	0.44	766	0.28
Denmark	Creamfinance	2605	0.48	650	0.24
Denmark	Mozipo Group	4	0.00	–	–
Denmark	Simbo	30,563	5.67	8258	2.99
Estonia	Capitalia	2	0.00	2	0.00
Estonia	Creditstar	7247	1.35	2498	0.90
Estonia	ESTO	3559	0.66	2998	1.09
Estonia	Mogo	1254	0.23	218	0.08
Estonia	Placet	763	0.14	588	0.21
Finland	BB Finance Group	9469	1.76	1186	0.43
Finland	Creditstar	1645	0.31	659	0.24
Latvia	AgroCredit	31	0.01	9	0.00
Latvia	Banknote	62,704	11.64	47,736	17.28
Latvia	Bino	64,000	11.88	14,330	5.19
Latvia	Capitalia	333	0.06	135	0.05
Latvia	Creamfinance	889	0.17	896	0.32
Latvia	Hipocredit	12	0.00	7	0.00
Latvia	Mogo	298	0.06	84	0.03
Latvia	Mogo Renti	300	0.06	268	0.10
Latvia	VIZIA	1754	0.33	3428	1.24
Lithuania	Capitalia	104	0.02	12	0.00
Lithuania	Hipocredit	8	0.00	4	0.00
Lithuania	Mogo	605	0.11	199	0.07
Lithuania	Mozipo Group	2452	0.46	456	0.17
Lithuania	Placet	1544	0.29	572	0.21
Poland	Alfakredyt	3588	0.67	3816	1.38
Poland	Capital Service	7263	1.35	345	0.12
Poland	Creamfinance Poland	18,528	3.44	19,232	6.96
Poland	Creditstar	27,166	5.04	13,671	4.95
Poland	Dziesiatka Finanse	894	0.17	105	0.04
Poland	Everest Finanse	31,382	5.83	10,317	3.73
Poland	Kuki	95,755	17.78	42,463	15.37
Romania	Credius	1122	0.21	–	–
Romania	Mikro Kapital Romania	13	0.00	11	0.00
Romania	Mogo	304	0.06	16	0.01
Romania	Mozipo Group	716	0.13	252	0.09
Spain	Creamfinance Spain	14,232	2.64	6806	2.46
Spain	Creditstar	19,323	3.59	6915	2.50
Spain	Dineo Credito	24,989	4.64	21,243	7.69
Spain	ID Finance	62,523	11.61	52,924	19.16
United Kingdom	Evergreen	5493	1.02	175	0.06
United Kingdom	Novaloans	8897	1.65	3658	1.32
United Kingdom	Peachy	426	0.08	–	–
**Total**		**538,656**	**100.00**	**276,230**	**100.00**

Total values are in bold

## Abbreviations

OECD: Organisation for Economic Co-operation and Development
SMEs: Small- and medium-sized enterprises
SSE: Shanghai Stock Exchange
WHO: World Health Organization
NPL: Non-performing loans
EU: European Union
EBA: European Banking Authority

## Footnotes

1 Refer to Table 2 for the description and breakdown of loans.

2 “Appendix 1” describes all the variables used in the study.

3 Please refer to “Appendix 2” for the breakdown of loans by loan originators.

4 We match these variables based on closing date or last payment date of each loan.

5 We classify loans that are more than 30 days late as ‘default’ loans.

6 We perform respective transformations of the marginal impact of coefficients based on logistic function.

7 Please, refer to “Appendix 2” for the distribution of the database by county and loan originators.

8 Stratified bootstrap samples based on loan originators and each month of 2020. Bootstrap sampling replications are conducted 1000 times for each regression estimations.

9 Late loans are further classified as: ‘1–15 days late’, ‘16–30 days late’, ‘31–60 days late’ and ‘60+ days late’.
